# *Clostridium* butyricum RH2 Alleviates Chronic Foot Shock Stress-Induced Behavioral Deficits in Rats via PAI-1

**DOI:** 10.3389/fphar.2022.845221

**Published:** 2022-04-06

**Authors:** Wenying Zhang, Tingyu Ding, Hong Zhang, Yuping Chen, Liping Liu, Jinjin Jiang, Siyuan Song, Hao Cheng, Changhao Wu, Jihu Sun, Qin Wu

**Affiliations:** ^1^ Jiangsu Vocational College of Medicine, Yancheng, China; ^2^ Marketing Department, Hangzhou Grand Biologic Pharmaceutical Inc., Hangzhou, China; ^3^ School of Biosciences and Medicine, University of Surrey, Guildford, United Kingdom

**Keywords:** *Clostridium* butyricum RH2, stress, mood disorders, gut-brain axis, hippocampus, BDNF

## Abstract

Recent investigations have demonstrated that the chronic stress-induced behavioral disorders can be ameliorated by probiotics including *Clostridium butyricum* (C. butyricum) via the gut-brain-axis. However, the molecular mechanisms underlying the beneficial effects of C. butyricum on brain remain largely unknown. Here, we investigated whether chronic foot shock stress (CFSS) paradigm used for a hypertensive animal model could induce mood disorders such as anxiety, depression and cognitive impairments. Then, we assessed the impact of *C. butyricum* RH2 on the behavior disorders and neurobiological alterations in the hippocampus. Male Sprague-Dawley (SD) rats received intermittent electric shocks for consecutive 14 days and were treated with *C. butyricum* RH2 for 17 days. Anxiety- or depression-like behaviors were evaluated by open field test (OFT), and elevated plus maze (EPM). The Morris water maze test (MWM) was used to evaluate the cognitive functions. CFSS intervention led to mild anxiety- or depression-like behavior or cognitive impairment and *C. butyricum* RH2 treatment reversed the CFSS-induced symptoms. The serum ACTH or CORT was increased following CFSS but was completely reversed by *C. butyricum* RH2 treatment. In the hippocampus of CFSS rats, the expressions of BDNF and TrkB were downregulated but proBDNF and P75^NTR^ were upregulated. These expression changes were partially reversed by *C. butyricum* RH2, suggesting a mode of action on BDNF and proBDNF balance. CFSS exposure resulted in downregulation of tissue-type plasminogen activator (tPA) but upregulation of plasminogen activator inhibitor 1(PAI-1), which could contribute to the decrease in BDNF by reduced conversion from proBDNF to BDNF in the hippocampus. *C. butyricum* RH2 treatment reversed the upregulated PAI-1 but not the downregulated tPA, which was in parallel with the amelioration of behavioral abnormalities, suggesting a novel tPA independent mechanism for PAI-1 action. Our results demonstrate for the first time that *C. butyricum* RH2 attenuates stress-induced behavior disorders via inhibiting the expression of brain PAI-1.

## 1 Introduction

Humans live in a stressful environment and are constantly exposed to unavoidable stressors. Different stressors can provoke a series of behavioral, emotional or physiological responses in animals (Gold, 2015). The stress-induced maladaptive responses are determined by the nature (physical or psychological), intensity and duration of the stressor. Electric foot shocks have been widely used for the development of various animal models of human disorders such as hypertension, anxiety, depression and post-traumatic stress disorder (PTSD) by introducing subtle variations in current intensity, duration, number of shock exposures and post-exposure treatment (Bali and Jaggi, 2015; Kim et al., 2017; Schöner et al., 2017). In the animal model of stress-induced hypertension, rats received intermittent electric shocks (Wu et al., 2020), but behavioral changes of rats exposed to this chronic foot shock stress (CFSS) paradigm remains unknown.

Chronic stress not only induces behavioral disorders, but also results in gut dysbiosis in animals and humans (Bharwani et al., 2016; Gao et al., 2018; Tian et al., 2020). Mounting evidence suggests that the gut microbiome is involved in mood disorders (Cruz-Pereira et al., 2020; Rutsch et al., 2020). Analysis of gut microflora in depressed patients or rodents has revealed a significant difference from that in healthy controls (Jiang et al., 2015; Zheng et al., 2016). Fecal microbiota transplantation from depressed patients induced depression-like behavior in the microbiota-deficient rodents and the depressed recipient animals exhibited disturbances of gut microbiota (Kelly et al., 2016; Zheng et al., 2016). Mice given the microbiota from the chronically stressed mice showed higher levels of anxiety- and depression-like behavior compared to the controls (Bruce-Keller et al., 2015; Li et al., 2019). Vice versa, fecal microbiota transplantation from the health donors to the stressed recipient rats ameliorated stress-induced depression-like behaviors (Rao et al., 2021). Based on these findings that gut dysbiosis is causally linked to the deleterious effects of stress (Cryan et al., 2020), restoration of gut microbiota homeostasis by probiotic intervention has gained a great attention in recent years (Rutsch et al., 2020). The beneficial effects of probiotics on the chronic stress-induced behavioral disorders, cognitive impairments, neurochemical abnormalities by reshaping the disturbed gut microbe in rodents have been shown by supplementation of Bifidobacterium breve CCFM1025(Tian et al., 2020), *Bifidobacterium breve* M2CF22M7 (Tian et al., 2019a), Bifidobacterium longum (Han and Kim, 2019), *Bifidobacterium longum* CCFM687 (Tian et al., 2019b), *Faecalibacterium prausnitzii* ATCC 27766 (Hao et al., 2019), *Lactobacillus helveticus* NS8 (Liang et al., 2015), *Lactobacillus casei* (Gu et al., 2020), *Lactobacillus kefiranofaciens* ZW3 (Sun et al., 2019), respectively. *Clostridium butyricum* (C. butyricum) is a butyric acid-producing Gram-positive anaerobe in the gut. The C. butyricum is a well-known probiotic and has been widely investigated for a potential usage in a wide range of human diseases (Stoeva et al., 2021). Recently, experimental studies have revealed that the administration of C. butyricum -specific strains including Miyairi 588 or WZMC1018 significantly improved depressive-like behavior in the chronically stressed mice through the gut-brain axis (Sun et al., 2018; Tian et al., 2019a). The mechanisms underlying the role of C. butyricum in alleviating the stress-induced psychiatric disorders include an increase of the 5-HT levels and BDNF expression in the hippocampus, suppression of pro-inflammatory cytokines and inhibition of hippocampal microglial activation (Sun et al., 2018; Tian et al., 2019a). In addition, C. butyricum can stimulate the secretion of intestinal GLP-1 which may stimulate the up-regulated GLP-1R in the hippocampus of the stressed mice (Sun et al., 2018). However, the antidepressive mechanism of C. butyricum remains largely unknown.

Brain derived neurotrophic factor (BDNF) plays an important role in learning and memory formation. Decreased expression of BDNF and upregulated proBDNF (precursor to BDNF) in brain have been implicated in the stress-induced mood disorders (Bai et al., 2016; Sun et al., 2018; Yang et al., 2020; Lin et al., 2021). BDNF can be converted from proBDNF by plasmin which is cleaved from plasminogen by tissue-type plasminogen activator (tPA). Plasminogen activator inhibitor 1(PAI-1) can inhibit tissue-type plasminogen activator (tPA), resulting in the accumulation of proBDNF. PAI-1 is widely expressed in the brain. The expression of PAI-1 was increased in the prefrontal cortex and hippocampus of the chronically stressed rats (Tang et al., 2015), suggesting that PAI-1 up-regulation may play a key role in the pathological process of depression (Tang et al., 2015; Jiang et al., 2016; Han et al., 2019).

*C. butyricum* RH2 has been found to exert anti-inflammatory effect through correction of gut microbiota dysbiosis induced by an antibiotic (Li et al., 2021). However, the beneficial role of *C. butyricum* RH2 in alleviating the stress-induced behavioral disorders or cognitive impairment and the underlying mechanisms has not yet been explored.

In the current study, we investigated whether the CFSS paradigm used for hypertensive animal model led to the development of behavioral disorders associated with anxiety, depression and cognitive impairments. Furthermore, we assessed the beneficial effects of *C. butyricum* RH2 on the behavior disorders and also examined whether *C. butyricum* RH2 exerted anxiolytic effects through decreasing the expression of PAI-1 in hippocampus of CFSS rats, aiming to explore a novel therapeutic approach for alleviation of the stress-induced mood impairments.

## 2 Materials and Methods

### 2.1 Animal Preparation

Male adult Sprague-Dawley (SD) rats (200–250 g) used in this study were purchased from the Shanghai Jiesijie Experimental Animal Co., Ltd. (SCXK (hu) 2018–0004). The rats were housed in a temperature-controlled room (22 ± 1°C) under a 12 h light-dark cycle with food and water ad libitum. All procedures were approved by the Animal Care and Use Committee of Jiangsu Vocational College of Medicine (Ethics review form NO.2019201).

Rats were randomly assigned into four groups: sham, stress, stress + *C. butyricum* RH2 (C.b) and positive control (Reserpine) groups, with 7 rats in each group. After 1 week of acclimatization, Stress + *C. butyricum* RH2 group rats were given *C. butyricum* RH2 (1 × 10^9^ CFU/ml/day/rat) by gastric gavage (*C. butyricum* RH2 was kindly shared by Chongqing Taiping Pharmaceutical Co., LTD., P. R. China) once a day for consecutive17 days (3 days before the stress and 14 days during the stress). *C. butyricum* RH2 solution was freshly prepared before administration. Freeze-dried bacteria powder of C. butyricum RH2 containing 4 × 10^9^ colony forming units (CFU)/g was adjusted in 4 ml sterilized saline to a final concentration of 1 × 10^9^ CFU/ml. The dose of *C. butyricum* RH2 used in the present study was based on previous studies (Sun et al., 2018; Li et al., 2021). Rats of stress group were given the same volume of saline by gastric gavage. On day 3, rats of stress + *C. butyricum* RH2 group and stress group were individually placed in a cage (22 cm × 22 cm×28 cm) with a grid floor and exposed to electric foot-shock combined with buzzer noise stress administered for 2 h twice a day, with a 4-h interval between the sessions, for consecutive 14 days (Wu et al., 2020). Control rats were also individually placed in a similar cage but without receiving stressors (electric shocks and noises). Then, behavioral tests were performed. After the behavioral experiments, blood and brain specimens of the experimental rats were taken out for further analysis. A single and repeated administration (once daily for 14 days) of reserpine ((Sigma-Aldrich, USA, 0.2 mg/kg i. p.) as the positive control group for depressive disorders (Antkiewicz-Michaluk et al., 2014).

Food intake was recorded every day throughout the experiment in each group of rats. Food consumed was recorded by subtracting the residual food from the food given (more than daily eaten) on the previous day. Body weight was monitored three times starting at the onset, on the 7th day and 14th day of CFSS. Body weight gain (Δ body weight) was calculated by subtracting the starting body weight from body weight on the 14th day.

**FIGURE 1:**
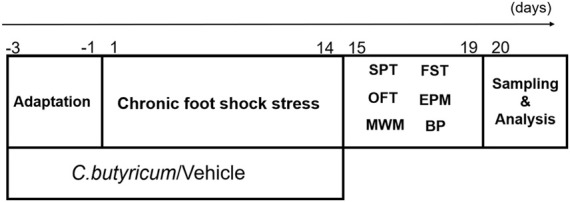
The schematic diagram of the experiments. SD rats were pretreated with *C. butyricum* RH2 or vehicle for 3 days before receiving chronic foot shock stress for 14 days, and the rats treated with *C. butyricum* RH2 or vehicle for 17 days in total. Between days 15 and 20, the rats were given behavioral tests (15th–16th day:SPT; 17th–19th day:FST, OFT, EPM; 15th–20th day:MWM). Blood and brain specimens were collected on the 20th day for further measurement and analysis. SPT, sucrose preference test; FST, forced swim test; OFT, open field test; EPM, elevated plus maze test; MWM, Morris water maze; BP, blood pressure.

The details of the experimental design conducted in this study are shown in Figure 1.

### 2.2 Behavioral Assessments

Behavioral alterations of CFSS rats were tested by the well established methods including sucrose preference test (SPT), forced swim test (FST), open field test (OPT), elevated plus maze test (EPM), and Morris water maze (MWM). The devices were provided by Anhui Zhenghua Biological Instrument Equipment Co. LTD. All behavioral tests were videotaped and data were analyzed by ANY-maze animal behavior analysis system developed by Stoeling, United States.

#### 2.2.1 Sucrose Preference Test

SPT was used for the measurement of anhedonic-like behavior. Before the experiment, rats were trained to drink sucrose solution (2% w/v) for 24 h. After being deprived of food and water for 12 h, each rat was given a bottle of sucrose solution and a bottle of drinking water for free intake. Two hours later, consumption of sucrose solution and drinking water were measured respectively. The animals’ sucrose preference (SP) was calculated as following formula: SP = sucrose solution consumed/ (sucrose solution consumed + water consumed) × 100%.

#### 2.2.2 Forced Swim Test

FST was used for the measurement of behavioral despair (giving up hope of escape). Rats were placed individually in a glass cylinder (50 cm in height and 20 cm in diameter) filled with 35 cm depth of water (23 ± 1°C). Before the experiment, the rats were put into the behavior laboratory to adapt to the environment for 1 h. During the test, each rat was gently placed in water and adapted for 2 min. Then, the sum of the time that the rat remained motionless in the water in the following 4 min was recorded. After the test, the rat was dried with a dry towel and placed in a warm place. After 20 min, it was moved back to its original cage. After 24 and 48 h, the rat received the second and the third test session, respectively. The average immobility time of the three trials was analyzed.

#### 2.2.3 Open Field Test

OFT was used to measure the spontaneous exploratory behavior and curiosity of animals to new environments. The apparatus for OFT was composed of a 50 × 50 × 40 cm^3^ open box. A camera attached to the recording system was placed above the box. Real-time images of rats were analyzed by the system to obtain the behavior data of the rats in open field. The bottom of the open box square was divided into nine squares of equal size, and the middle one was defined as the central area. Before the experiment, the rat was put into the behavior laboratory to adapt to the environment for 1 h. In a quiet environment, the rat was put into the central square at the bottom of the open field and left to move freely for 5 min. The following indexes were observed and recorded: the total movement distance, the movement distance in central area and average movement speed. The box was cleaned with 75% alcohol and dried between the tests. The environment remained quiet during the entire procedure.

#### 2.2.4 Elevated Plus Maze Test

EPM is often used to assess an animal’s anxiety or exploratory behavior in a new environment. The experimental device was elevated 70 cm from the ground, and was composed of two open arms (122.3 cm) and two closed arms (110.5 cm) with the central area (12 × 12 cm^2^). It was perpendicular to each other in a cross shape of “+.” A camera was placed above the joint between the open arm and the closed arm. The experiment was performed in a dim light environment. Before the experiment, the rats were put into the behavior laboratory to adapt to the environment for 1 h. At the beginning of the experiment, the rat was placed at the central junction with the head facing an open arm, and allowed to move freely for 5 min. When all four legs of the rat entered the open/closed arm, one entry was recorded. The percentage of times the rats entered the open arm and the percentage of time they stayed in the open arm were calculated to evaluate the emotional state of the rats. Between the experiments, each rat was cleaned of residues in the box to remove the smell of the previous rat, so as not to affect the behavior of the next rat.

#### 2.2.5 Morris Water Maze

The Morris water maze test was performed to assess the memory and spatial learning functions of animals. A hidden platform with a diameter of 12 cm was maintained in II quadrant of a circular basin, 1–2 cm below the surface of water (23 ± 1°C). The place navigation task lasted for 5 days. Rats were placed into the water from four entry points facing the wall every day, and the time of finding the platform (escape latency) was recorded. In addition to observing and recording the escape latency, the swimming track, total swimming distance and average swimming speed in the water were also observed and recorded. After the positioning navigation experiment, spatial probe test was performed to assess memory consolidation by removing the platform. Each rat was placed into the water from four different quadrants. The crossing number of the former platform position and time spent crossing the former platform quadrant were recorded in a period of 60 s.

### 2.3 Western Blot

The protocol of western blot was the same as that previously reported [Wu et al., 2020]. Briefly, 200 μl of pre-cooled extraction reagent was added to the rat Hip sample. The extraction reagent was pre-added with 2 μl of protease inhibitor, 10% PMSF, and phosphatase inhibitor. Protein concentration was measured by the BCA method. 10% separation gel and 60 V constant voltage were first used, then 120 V constant voltage was used for electrophoresis until the bromophenol blue color reached the bottom of the separation gel. Subsequently, proteins were transferred onto a PVDF membrane that was washed with TBST solution 3 times, 5 min each time. The membrane was then blocked with 5% milk in PBS-Tween 20 for 1 h, and incubated overnight at 4°C with mouse anti-GFAP (3670s, Cell Signaling Technology, United States; 1:1,000), rabbit anti-BDNF (ab108319, Abcam, United Kingdom; 1:1,000), rabbit anti-TrkB (ab33655, Abcam, United Kingdom; 1:1,000), rabbit anti-p75^NTR^ (ab52987, Abcam, United Kingdom; 1:1,000), anti-Pro-BDNF (sc-65513, SANTA CRUZ, USA; 1:200), mouse anti-β-Actin (AF0003, Beyotime, China; 1:1,000) antibodies, rabbit anti-PAI-1 (ab66705, Abcam, United Kingdom; 1:1,000) and rabbit anti-tPA (10147-1-AP, Proteintech, China, 1:1,000), respectively. Then, the membrane was washed 3 times (10 min each time), incubated with anti-rabbit or anti-mouse secondary antibodies at room temperature for 60 min, washed, immersed in ECL solution for about 1 min, and photographed with a gel imaging system. The bands were quantified by ImageJ software.

### 2.4 Immunofluorescence Staining

The rat brain samples were fixed (4% paraformaldehyde, PFA), gradient sugar deposition (10, 20, 30% sucrose), and then coronal sections (35 mm in thickness) of the Hip were sliced using a freezing microtome (CM 1850; Leica, Germany). Sections were incubated in rabbit anti-PAI-1 (ab66705, Abcam, United Kingdom; 1:200) and rabbit anti-tPA (10147-1-AP, Proteintech, China, 1:50), 4°C overnight. After the sections were washed with 0.01 PBS, they were incubated with goat anti-mouse IgG H&L (Alexa Fluor^®^ 488) (ab150113, Abcam, United Kingdom; 1:200) or goat anti-rabbit IgG H&L (Cy3 ^®^) preadsorbed (ab6939, Abcam,United Kingdom; 1:200) as secondary antibody at room temperature for 2 h. A laser scanning confocal microscope (LSM 900, Zeiss, Germany) was used to observe the positive cells.

### 2.5 Enzyme-Linked Immunosorbent Assay

After the rats were decapitated, blood samples were collected, centrifuged, and the serum was stored at −20°C. The serum norepinephrine (NE), adrenocorticotropic hormone (ACTH) and corticosterone (CORT) levels were measured using an ELISA kit (Weiao, Shanghai, China). All samples and standards were measured in duplicate.

### 2.6 Statistical Analysis

Data are presented as mean±the standard error of the mean (SEM). All statistical analysis was performed using GraphPad Prism 8.0, compared by one-way ANOVA followed by the Tukey post hoc test. *p* < 0.05 was considered statistically significant.

## 3 Results

### 3.1 C. butyricum RH2 Treatment Improved CFSS-Induced Anxiety- and Depression-like Behaviors

**FIGURE 2:**
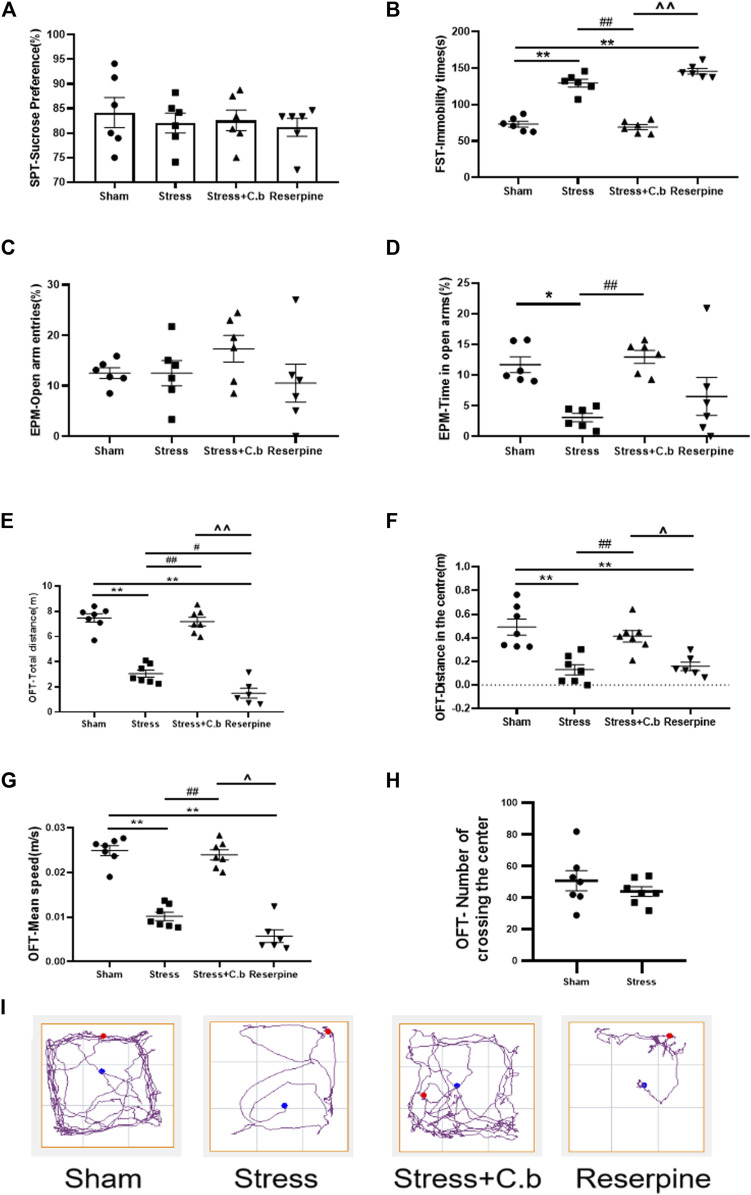
C. butyricum RH2 treatment improved chronic foot shock stress (CFSS)-induced behavioral deficits in rats. **(A)** CFSS treatment did not induce a decrease in the sucrose consumption. **(B)** C. butyricum RH2 improved the increased immobility time induced by CFSS. **(C)** CFSS treatment did not change open arm entries. **(D)** C. butyricum RH2 prolonged CFSS-induced decrease in time spent in the open arm time. **(E–G)** C. butyricum RH2 lengthened the central area distance, the total distance and the average speed in CFSS rats. **(H)** The number of crossing the center area in the OFT was not significantly different between CFSS rats and control rats (*p* > 0.05). **(I)** Typical track in OFT. The data are expressed as mean ± SEM (n = 7 in sham, stress, stress + C. butyricum group; n = 6 in reserpine group). *p < 0.05, **p < 0.01 versus sham group; #*p* < 0.05, ##*p* < 0.01 versus stress group; ^*p* < 0.05, ^^*p* < 0.01 versus stress + C. butyricum group.

The effects of chronic administration of C. butyricum RH2 on anxiety-like behaviors were evaluated in open field test (OFT), and elevated plus maze (EPM). Compared to rats in the sham group, anxiety-like behaviors were significantly induced in rats subjected to CFSS, as reflected by a decrease in the percentage time spent in open arm in the EPM (Figure 2D, *p* < 0.05), and the reduction in total distance, central distance or mean speed in the OFT (Figures 2E–G, *p* < 0.01). The number of events crossing the center area in the OFT was not significantly different between CFSS rats and control rats (Figure 2H, *p* > 0.05), suggesting that CFSS did not affect the locomotor activity. Rats exposed to CFSS also exhibited significant depression-like behaviors, as reflected by the increased immobility time in the forced swim test (FST) (Figure 2B, *p* < 0.01). However, CFSS exposure was without effects on the percentage of open arm entries in the EPM test (Figure 2C, *p* > 0.05) and sucrose consumption in the sucrose preference test (SPT) (Figure 2A, *p* > 0.05). Seventeen days of C. butyricum RH2 treatment reversed the CFSS-induced anxiety-like symptoms (Figure 2D, *p* < 0.01; Figures 2E–G, *p* < 0.01; Figure 2H, *p* < 0.01) and the depression-like behaviors (Figure 2B, *p* < 0.01). Reserpine was used as positive control and reserpine treatment induced anxiety-and depression-like behaviors as expected, which is in line with CFSS exposure. Typical tracks in OFT in sham-, stress-, stress + C. butyricum RH2—and reserpine treated animals are shown in Figure 2I, respectively.

### 3.2 C. butyricum RH2 Treatment Ameliorated CFSS-Induced Cognitive Deficits

**FIGURE 3:**
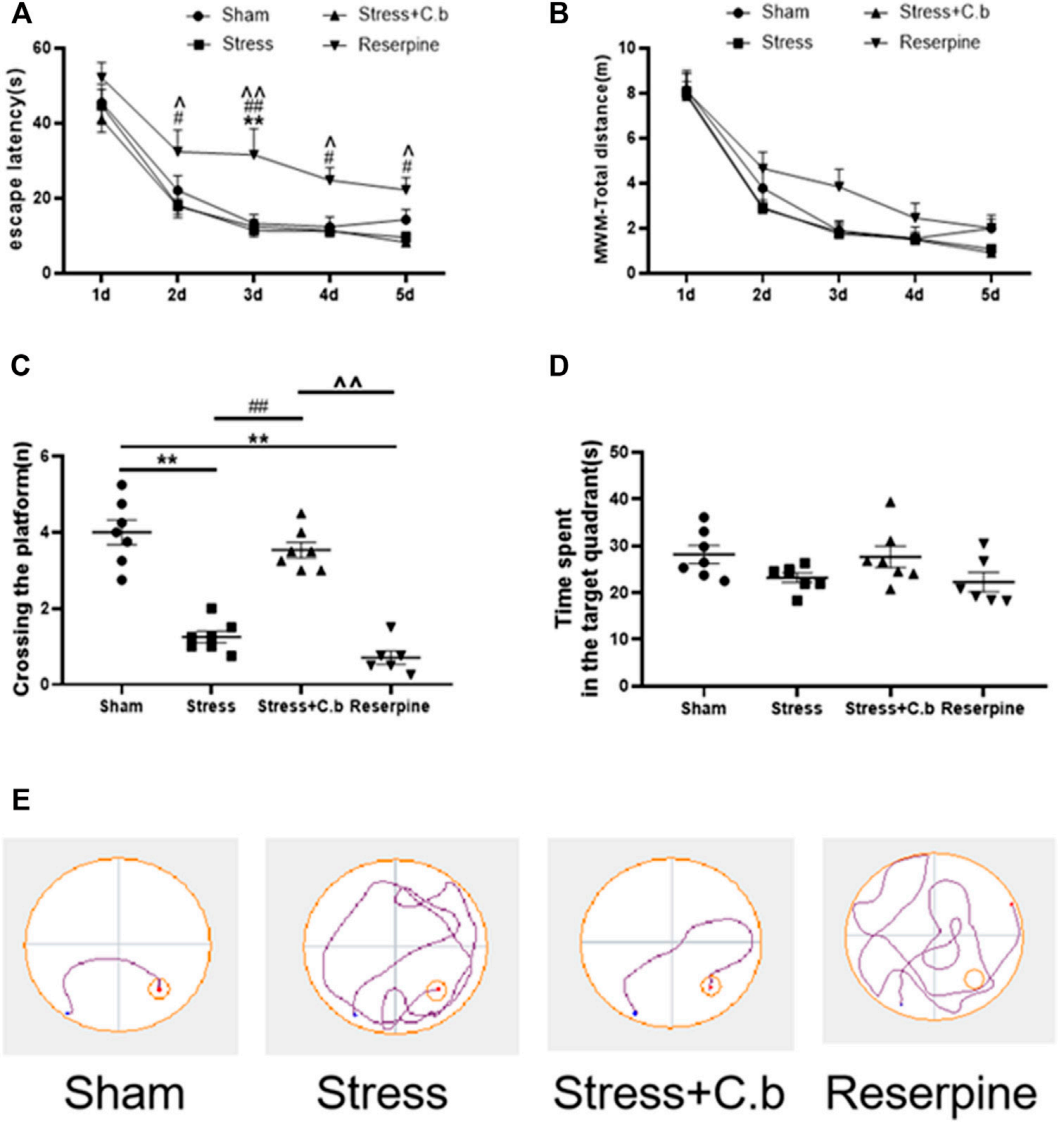
C. butyricum treatment ameliorated the cognitive deficits in the CFSS rats by reversing the decreased number of crossing in target quadrant **(C)** but revealed no effects on the escape latency **(A)**, the total distance **(B)** and time spent in the target-quadrant **(D)**. **(E)** The representative locus plot in the MWM test. The data are expressed as mean ± SEM (n = 7 in sham, stress, stress + C. butyricum group; n = 6 in reserpine group). *p < 0.05, **p < 0.01 versus sham group; #*p* < 0.05, ##*p* < 0.01 versus stress group; ^*p* < 0.05, ^^*p* < 0.01 versus stress + C. butyricum group.

The Morris water maze test was carried out for 5 days to evaluate the effects of 17 days C. butyricum RH2 treatment on the cognitive functions. During the five consecutive training days, all rats learned to find the submerged platform and the escape latency were gradually shortened. Two-way ANOVA of the escape latency revealed no significant difference between sham, CFSS and CFSS + C. butyricum groups (Figure 3A, *p* > 0.05) but a significantly longer escape latency in reserpine group (Figure 3A, *p* < 0.05 or 0.01, respectively). Two-way ANOVA of the total escape distance revealed no significant difference between any of the groups (Figure 3B, *p* > 0.05). In the probe trails, the number of platform crossings in CFSS treated rats was significantly decreased compared to that in sham rats and the decrease was significantly reversed by C. butyricum RH2 treatment (Figure 3C, *p* < 0.01). The time spent in the target quadrant was not significantly different between the groups regardless of a trend of decline in CFSS or reserpine groups (Figure 3D, *p* > 0.0.5). The representative locus plots of Figures 3A,B are shown in Figure 3E.

### 3.3 Effects of C. butyricum RH2 Treatment on Weight Gain and Food Intake of CFSS Rats

**FIGURE 4:**
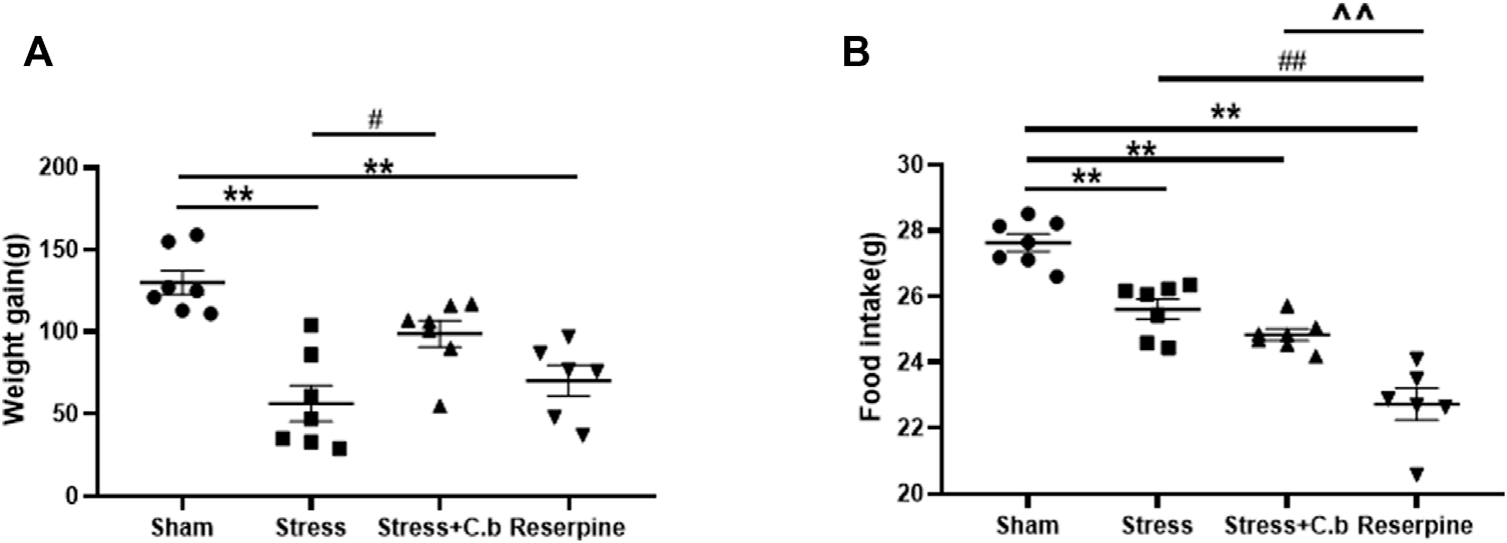
C. butyricum RH2 treatment reversed CFSS-induced decrease in weight gain but not the decrease in food intake. **(A)** The weight gain **(B)** The food intake. **p < 0.01 versus sham group; #*p* < 0.05, ##*p* < 0.01 versus stress group; ^^*p* < 0.01 versus stress + C. butyricum group.

Body weight gain was significantly different between groups as shown in Figure 4A (*p* < 0.01). After 2 weeks of CFSS exposure, the body weight gain of rats was found to be significantly reduced compared to controls (*p* < 0.01). The decrease was significantly reversed by C. butyricum RH2 treatment for 17 days (*p* < 0.05). The measurement of food intake exhibited a significant difference between the groups as well (Figure 4B, *p* < 0.01). In comparison with sham rats, however, C. butyricum RH2 treatment could not reverse the decrease of food intake (*p* > 0.05). Reserpine remarkably reduced body weight gain and food intake of rats.

### 3.4 Effects of C. butyricum RH2 Treatment on Serum ACTH or CORT

**FIGURE 5:**
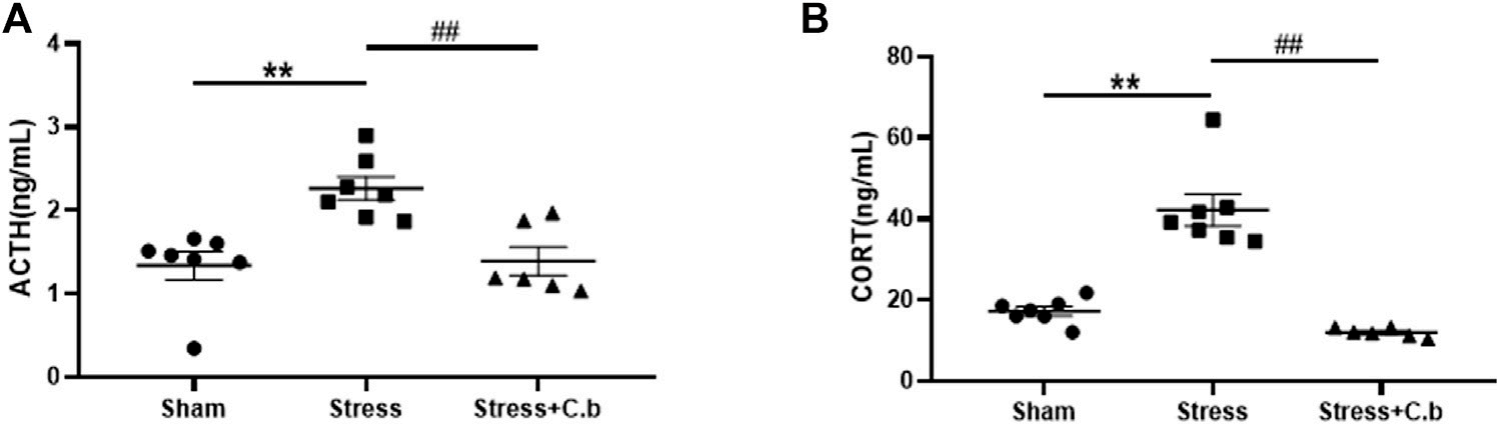
C. butyricum RH2 treatment reversed CFSS-induced decreases in serum ACTH and CORT. **(A)** The ACTH **(B)** The CORT. **p < 0.01 versus sham group; ##*p* < 0.01 versus stress group.

Measurements using ELISA revealed that the serum levels of both ACTH and CORT were significantly increased (Figures 5A–B, *p* < 0.01) in CFSS rats compared to the control rats. After 17 days of C. butyricum RH2 treatment, the increase of the serum ACTH or CORT levels in CFSS rats was reversed (Figures 5A–B, *p* < 0.01).

### 3.5 Effects of C. butyricum RH2 Treatment on Expression of BDNF, proBDNF, TrkB and P75^NTR^ in Hippocampus

**FIGURE 6:**
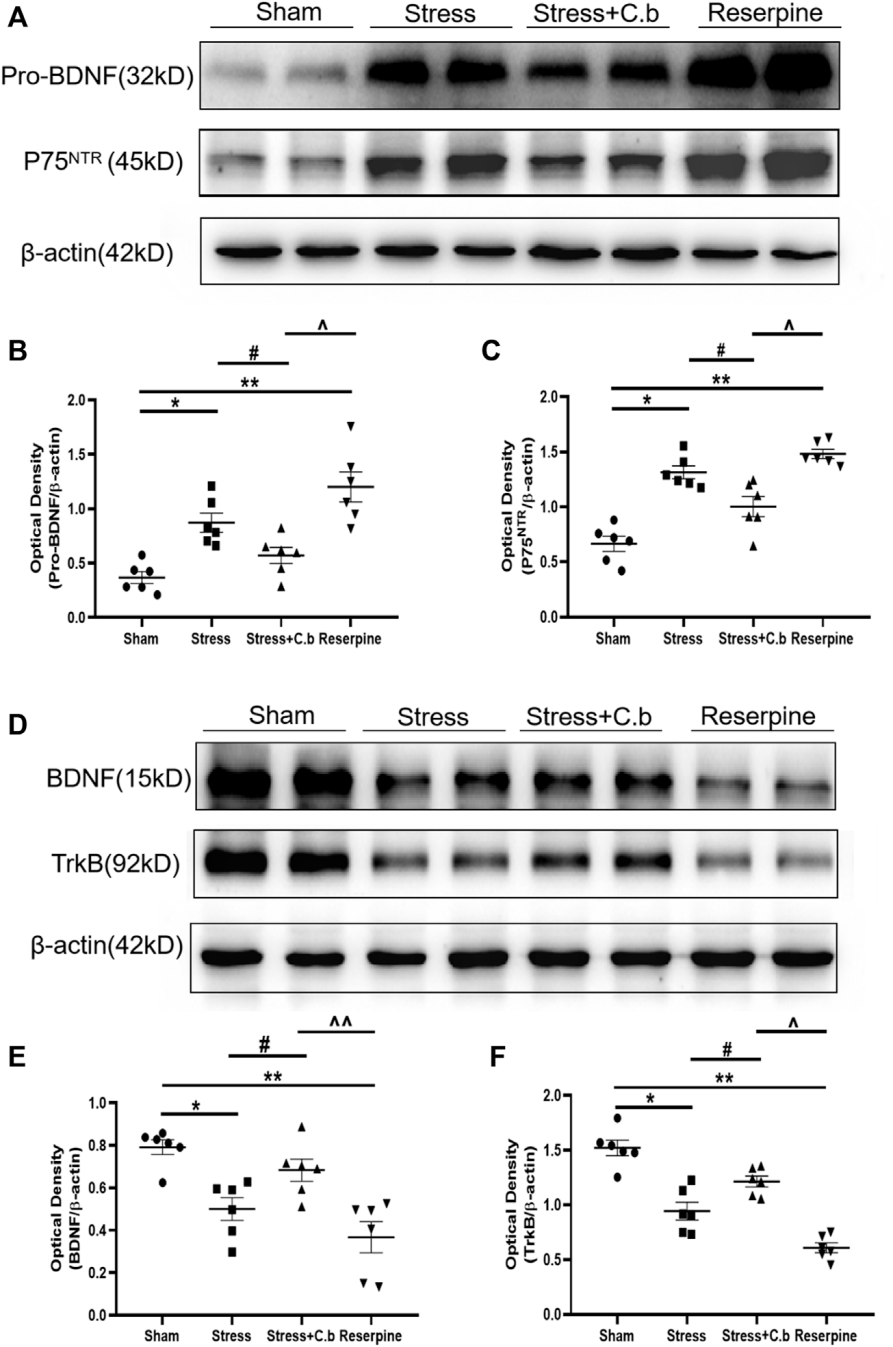
Effects of C. butyricum RH2 on the expression of proBDNF, P75^NTR^, BDNF and TrkB in the hippocampus of CFSS rats. **(A–C)** proBDNF and P75^NTR^ were upregulated after CFSS but the increase was partially reversed by C. butyricum RH2. **(D–F)** BDNF and TrkB were downregulated after CFSS but the decrease was partially reversed by C. butyricum RH2. *p < 0.05, **p < 0.01 versus sham group; #*p* < 0.05, ##*p* < 0.01 versus stress group; ^*p* < 0.05, ^^*p* < 0.01 versus stress + C. butyricum group.

The mechanisms for CFSS induced neurological changes and the C. butyricum RH2 action were further sought. As shown by western blotting analysis in Figures 6A–C, CFSS significantly increased protein levels of proBDNF and P75^NTR^ (receptor for pro-BDNF) in the hippocampus (*p* < 0.05), whereas C. butyricum RH2 treatment reversed these changes (*p* < 0.05), i.e. decreasing the upregulated expression levels of proBDNF and P75^NTR^. However, their respective protein levels in the CFSS + C. butyricum group were still significantly higher than those in the sham group. These data, indicate that C. butyricum RH2 treatment only partially reversed CFSS-induced upregulation of proBDNF and P75^NTR^. On the contrary, CFSS significantly decreased protein levels of BDNF and TrkB (receptor for BDNF) in the hippocampus (Figures 6D–F, *p* < 0.05), whereas C. butyricum RH2 treatment partially reversed CFSS-induced downregulation of both BDNF and TrkB (Figure 6B, *p* < 0.05). These data together were consistent with a mechanism of changed balance between proBDNF and BDNF in the action of C. butyricum RH2. Finally, these CFSS-induced changes in protein levels of proBDNF, P75^NTR^, BDNF, TrkB were also seen in reserpine intervention, emphasizing stress-specific effects.

### 3.7 Effects of C. butyricum RH2 Treatment on Expression of PAI or t-PA

**FIGURE 7:**
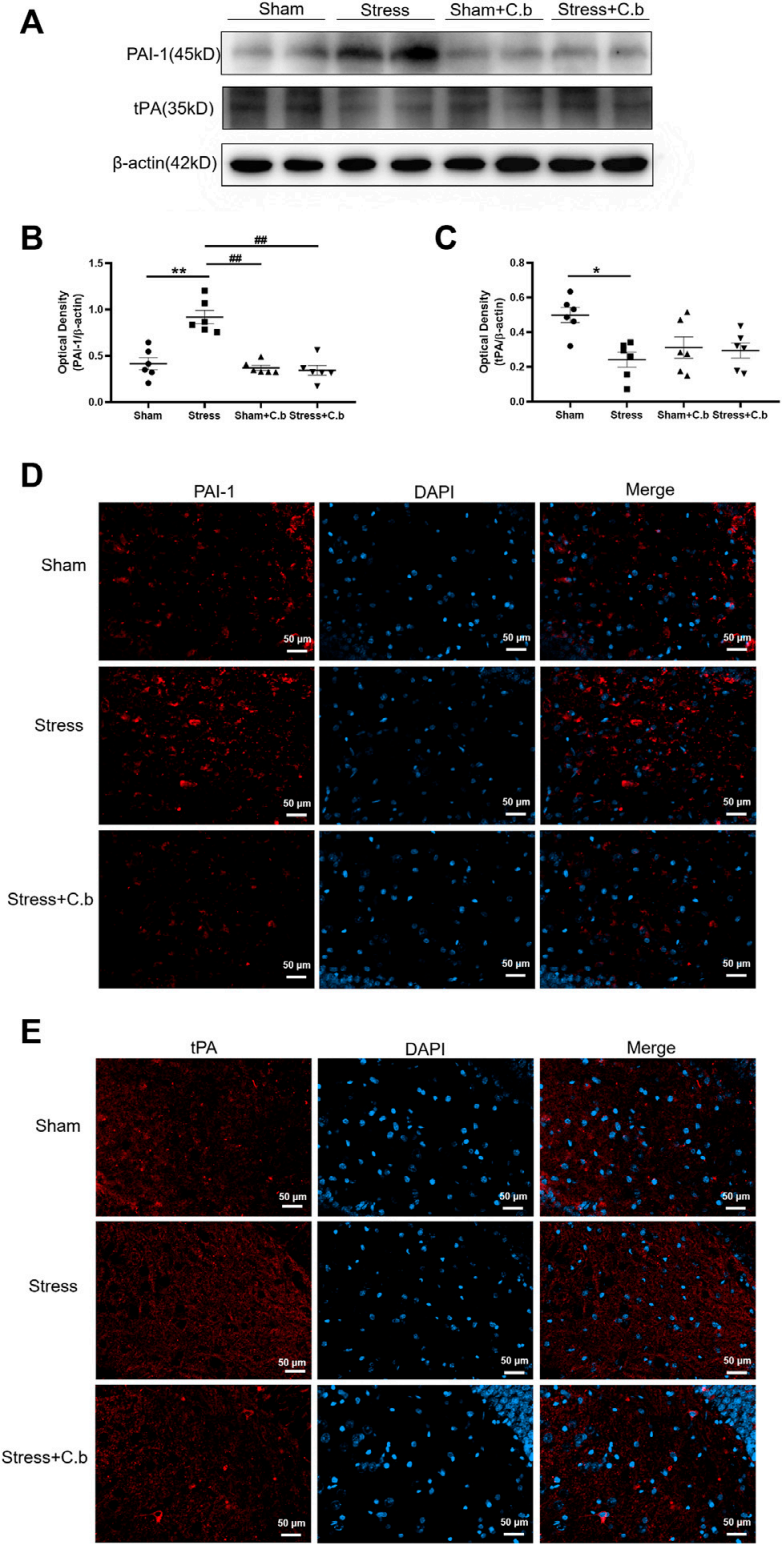
C. butyricum RH2 treatment reversed CFSS-induced upregulation of PAI-1 in the hippocampus. **(A–C)** Western blot analysis of expression of PAI-1 and tPA in the hippocampus of CFSS rats before and after C. butyricum RH2 treatment. **(D–E)** The immunoreactive spots of PAI-1 and tPA protein in the hippocampus of CFSS rats before and after C. butyricum RH2 treatment. *p < 0.05, **p < 0.01 versus sham group; #*p* < 0.05, ##*p* < 0.01 versus stress group; ^*p* < 0.05, ^^*p* < 0.01 versus stress + C. butyricum group.

To explore the molecular basis underlying the effects of C. butyricum RH2 treatment on the expression of proBDNF and BDNF as seen in Figure 6, the protein levels of tPA (a proteolytic enzyme for cleavage of proBDNF to mBDNF) and PAI-1 (the tPA inhibitor) were examined respectively. Western blot analysis of PAI-1 immunoreactivity revealed a low protein level of PAI-1 in sham rats but the protein level of PAI-1 was significantly increased after CFSS exposure (*p* < 0.01, Figures 7A,B). The CFSS-induced increase of PAI-1 was completely reversed by C. butyricum RH2 treatment (*p* < 0.01, Figures 7A,B) whereas C. butyricum RH2 alone had no effects on PAI-1 expression in sham rats (*p* > 0.05, Figures 7A,B). In contrast to PAI-1 changes, the expression of tPA protein was significantly downregulated by CFSS exposure (*p* < 0.05, Figures 7A,C). However, the CFSS-induced decrease of tPA protein expression was not reversed by C. butyricum RH2 treatment (*p* > 0.05, Figures 7A,C). The immunoreactive spots of PAI-1 and tPA protein examined by LCSM in hippocampal sections exhibited the same changes as that detected by the western blot analysis, respectively (Figures 7D,E). These data suggest a key role for PAI-1 in the reversal action of C. butyricum RH2 on CFSS induced pathological changes, but it is tPA-independent.

## 4 Discussion

In the present study, we demonstrated that the CFSS paradigm induced not only a physiological response as indicated by the elevation of blood pressure (Wu et al., 2020a; Wu et al., 2020b) but also behavioral changes, as reflected by mild anxiety or depression-like behavior. The present data support the idea that the CFSS is a complex stressor with both physical and emotional components (Bali and Jaggi, 2015). Moreover, CFSS resulted in mild cognitive impairment as evaluated by the Morris water maze test. However, mild electric foot shocks of 0.15 mA intensity for 0.5 s induced cognitive enhancement in mice (Bali et al., 2013). In the CFSS paradigm, the output voltage instead of current was used. The amplitude of the output voltage was adjusted just enough to cause the rat’s forelimbs to cling to the side wall of the cage in an upright position. These results suggest that varying shock parameters including current/voltage intensity, duration, number, and interstimulus interval provoke differential stress responses in animals (Bali and Jaggi, 2015).

The CFSS paradigm has been employed to induce a hypertensive model in rodents (Wu et al., 2020). Since the electric foot shock induced stress has been a well established animal model of mood disorders, especially PTSD (Bali and Jaggi, 2015; Kim et al., 2017; Schoner et al., 2017), we question whether the CFSS paradigm influences psychological behavioral alterations. In this study, CFSS exposure induced a significant decrease in food consumption and body weight gain. The decreased food intake and body weight gain was a well described symptom of exposure to chronic stress (Yau and Potenza, 2013; González-Torres and Dos Santos, 2019). Results of EPM, and OFT exhibited anxiety-like behavior in CFSS rats, but the percentage of open arm entries in EPM test was not significantly decreased in the stressed rats. The lack of the influence of CFSS on the percentage of open arm entries suggests that CFSS exposure did not produce a stable anxiety-like behavior in terms of EPM test. The SPT and FST were used to detect depression-like behavior. The decrease in sucrose consumption in SPT indicates anhedonia, a symptom of depression. In this study, CFSS exposure did not induce a significant decrease in sucrose consumption/preference. These findings were in accordance with previous studies, suggesting that simple tests of sucrose consumption may not be valid as a hedonic measure in the chronic stress-induced depression models (Matthews et al., 1995; Forbes et al., 1996). While no inter-group differences in sucrose consumption, the increased immobility time in FST was induced in rats exposed to CFSS. This reflects depression-like behaviors because the behavioral outcome of the normalized FST has proven to be a reliable marker of depression (Cryan et al., 2005). In the Morris water maze test, CFSS subjected rats displayed significantly fewer platform crossings than the controls. But the escape latency, the total escape distance, and the time spent in the target quadrant were not significantly different. The current evidence of the Morris water maze test suggests a slight cognitive impairment induced by CFSS. Taken together with the behavioral variations described above, the CFSS paradigm used for the hypertensive animal model has mild effects on mood behavior and cognition but could not be employed as a valid depression model. Unlike the widely used depression models including chronic unpredictable mild stress (CUMS) or chronic mild stress (CMS) with more than 4 weeks of duration in their paradigms, the duration of the CFSS paradigm is 2 weeks. A 2-week duration in CFSS paradigm is enough to induce a stable sympathetic excitation which in turn induced a sustained elevation of blood pressure (Wu et al., 2020), but might not be enough to produce a stable depression model.

Considering stress-induced behavioral impairment is at least partially a consequence of gut microbiota alteration, supplementation with probiotics has been considered as a potential but promising therapy to counteract the detrimental effects of stress by restoring the microbiota (Foster et al., 2017; Nishida et al., 2019; Tian et al., 2020; Westfall et al., 2021). Our observation revealed that *C. butyricum* RH2 treatment ameliorated the CFSS-induced anxiety/depression-like symptoms, cognitive decline and the decrease in body weight gain but not in food intake. The beneficial behavioral effects of *C. butyricum* RH2 are in line with the previous studies in CUMS mice treated with the other *C. butyricum*-specific strains (Tian et al..2020; Sun et al., 2018). The probiotic C. butyricum provides benefit to the microbial ecosystem of the gut by increasing probiotics and decreasing pathogens (Zhao et al., 2020), inducing a higher abundance of *Clostridium* at the genus level which was positively correlated with the attenuation of the stress-induced behavioral disorders (Tian et al., 2019b) and producing more butyrate as the C. butyricum genome contains butyrate producing gene buk (butyrate kinase) (Louis et al., 2004; Stoeva et al., 2021). Our previous studies revealed that the CFSS paradigm altered gut microbiota characterized by decreases in the richness or diversity and the species composition of gut microbiota (Wu et al., 2020a). The *C. butyricum* RH2 has been proven to restore the disturbance of α-diversity and β-diversity of gut microbiota in ceftriaxone-treated mice (Li et al., 2021). Moreover, our unpublished data showed that the CFSS paradigm decreased number of butyric acid-producing bacteria. The abundance of the bacteria was significantly lower in CFSS rats than in control rats. Of the reduced bacterial units—210 OTUs, 25 were related to the butyrate producing ability. Furthermore, the butyric acid of feces was significantly lower in CFSS rats than in control rats. *C. butyricum* commonly exists in the gut of humans and animals, producing butyric acid (Detman et al., 2019), which has been implicated in depression (van de Wouw et al., 2018). Exposure to psychosocial stress decreased the relative abundance of *Clostridium spp*. in the caecum of mice (Bailey et al., 2011). *C. butyricum* is one of the commonly used butyrate-producing bacteria in clinical settings. In the present study, supplementation of *C. butyricum* RH2 reversed the decrease in butyric acid-producing bacteria and butyric acid in CFSS rats (unpublished data), which might mediate the beneficial role of *C. butyricum* RH2 in amelioration of mood deficits of CFSS rats. Our current investigation adds additional evidence that the probiotics supplementation can ameliorate stress related mood changes by rectifying dysbiosis and restoration of the normal balance in the gut microbiome.

It is well documented that chronic stress results in hyperactivation of the HPA axis. Increasing evidence suggests that the imbalances of the HPA axis could be the pathogenesis of the stress-induced behavioral aberrations (Lin et al., 2016; Huo et al., 2017; Kinlein et al., 2019). Recent studies have demonstrated that gut microbiota modulates stress-related behavior disorders via the HPA axis (Frankiensztajn et al., 2020; Misiak et al., 2020). This is supported by previous studies showing that probiotics supplementation attenuates the stress response by modulating the HPA axis activity (Ait-Belgnaoui et al., 2018). We previously reported that CFSS paradigm led to overactivation of the HPA axis and the exacerbated HPA axis was causally linked to CFSS-induced gut dysbiosis (Wu et al., 2020b). In the present study, the increased serum levels of ACTH and CORT in CFSS rats were almost completely reversed by *C. butyricum* RH2 intervention. The normalization of hormones ACTH and CORT by *C. butyricum* RH2 was accompanied by alleviation of behavioral disorders, providing evidence that beneficial effects of *C. butyricum* RH2 on stress-induced maladaptive changes may be achieved by improving the HPA axis dysfunctions via reshaping the gut microbiota.

Neurotrophin BDNF, by binding with high affinity to the tropomycin receptor kinase B (TrkB) receptor, is vital for synaptic formation, synaptic plasticity in the brain. The numerous studies suggest that BDNF-TrkB signaling plays an important role in learning, memory formation, and the pathophysiology of mood disorders (Vicario-Abejón et al., 2002; Autry and Monteggia, 2012; Duman et al., 2021). Clinical studies in major depressive disorder patients have shown that BDNF and TrkB levels were downregulated in the hippocampus and frontal cortex (Pandey et al., 2008; Thompson et al., 2011; Autry and Monteggia, 2012). An increase in brain BDNF levels by direct infusion or transgenic overexpression of BDNF-TrkB signaling in the hippocampus produces antidepressant-like behavioral effects (Shirayama et al., 2002; Hoshaw et al., 2005; Govindarajan et al., 2006). These data suggest a causal link between BDNF in the hippocampus and depression-like behavior. The central reduction in BDNF and its receptor TrkB has also been found in depressive animals subjected to various stressors (Phillips, 2017; Sun et al., 2018; Duman et al., 2021). Consistently, the expression of BDNF and TrkB was significantly downregulated in the hippocampus of CFSS rats in the present study. The selective deletion of BDNF in the hippocampus does not induce an increase in depression-like behavior per se but attenuates the responses of the antidepressant (Adachi et al., 2008), suggesting that the downregulated endogenous BDNF-TrkB signaling in the hippocampus of CFSS rats may not contribute to CFSS-induced anxiety/depression-like behaviors but to the susceptibility to mood disorders (Adachi et al., 2008). Gut dysbiosis downregulates the expression of BDNF and TrkB in the hippocampus (Bercik et al., 2011; Bistoletti et al., 2019). Therefore, the decreased BDNF and TrkB in the hippocampus of CFSS rats may be attributable to the gut dysbiosis induced by CFSS paradigm (Wu et al., 2020a). C. butyricum supplementation has been found to restore the decreased brain BDNF levels after stress (Sun et al., 2018) or sepsis (Liu et al., 2020). Given the role of *C. butyricum* RH2 in reshaping gut microbiota (Li et al., 2021), we assessed the effect of *C. butyricum* RH2 treatment on the expression of BDNF in the hippocampus of CFSS rats. Our results reveal that *C. butyricum* RH2 administration partially reversed the decrease in BDNF, which was in parallel with the amelioration of behavioral abnormalities. This demonstrates the involvement of upregulation of BDNF in the anxiolytics effect of *C. butyricum* RH2, at least in part.

BDNF is the cleaved form of proBDNF. Being a precursor to BDNF, proBDNF also binds specifically to the p75 neurotrophin receptor (p75^NTR^) to exert opposite biological effects to BDNF (Greenberg et al., 2009). In contrast to the downregulation of BDNF, the expression of proBDNF and p75^NTR^ was increased in the hippocampus of CFSS rats, which is in line with previous studies in other stressed rodents (Bai et al., 2016; Yang et al., 2020; Lin et al., 2021). Mounting evidence from patients and animals suggests a close association of proBDNF and p75^NTR^ with depression. In major depressive disorder patients, serum proBDNF and p75^NTR^ were significantly increased (Zhou et al., 2013). In animal models of depression, proBDNF and p75^NTR^ were upregulated in the brain areas such as hippocampus. Intracerebroventricular injection of proBDNF triggered depression-like behavior in normal rats (Bai et al., 2016). The upregulated proBDNF/ P75^NTR^ in the brain has been implicated in the pathogenesis of stress-induced mood disorders (Bai et al., 2016; Yang et al., 2020; Lin et al., 2021). Suppression of proBDNF/ p75^NTR^ signaling alleviated depressive and anxiety-like behaviors in chronically stressed mice (Yang et al., 2017; Lin et al., 2021). In CFSS rats, CFSS-induced upregulation of proBDNF/p75^NTR^ signaling was partially reversed by *C. butyricum* RH2 treatment, which was associated with the attenuation of CFSS-related mood disorders. In line with previous studies (Tian et al., 2020; Yang et al., 2020), these results indicated that CFSS elicited the imbalance between the proBDNF/p75^NTR^ and BDNF/TrkB signaling pathways. The *C. butyricum* RH2 corrected this imbalance in part, accounting to some extent for the beneficial effect of *C. butyricum* RH2 on mood disorders after CFSS. However, the molecular mechanisms by which *C. butyricum* RH2 normalized the imbalance remain to be explored.

CFSS resulted in the upregulation of proBDNF and downregulation of BDNF in the hippocampus, both of which could potentially synergistically elicit depression- and anxiety-like behaviors. To increase conversion of proBDNF to BDNF within the hippocampus would counter the deleterious effects of CFSS. In accordance, we speculated that *C. butyricum* RH2 restored CFSS-induced changes in proBDNF and BDNF by enhancing pro-BDNF cleavage into mature BDNF. Extracellular proBDNF can be converted to mature BDNF by plasmin (Pang et al., 2004). Plasmin is cleaved from plasminogen by tissue-type plasminogen activator (tPA) (Melchor and Strickland, 2005). The downregulation of tPA or decrease in tPA activity may decrease proBDNF cleavage, resulting in an increase of proBDNF levels and decrease of BDNF levels. Plasminogen activator inhibitor 1(PAI-1) is the major endogenous inhibitor for tPA (Melchor and Strickland, 2005). PAI-1 may decrease proBDNF cleavage and increase proBDNF levels by inhibiting the tPA/plasminogen system. Thus, the role of PAI-1 in the proBDNF and BDNF balance via tPA regulation under CFSS was explored. In this study, the protein level of PAI-1 in the hippocampus of CFSS rats was greatly increased while tPA was significantly decreased, accounting for the upregulation of proBDNF and downregulation of BDNF. Peripheral PAI-1 expression is increased by corticosterone treatment and PAI-1 deficiency significantly reduces GC-induced deleterious effects such as insulin resistant, bone loss (Tamura et al., 2015). This means that the CFSS-induced increase in CORT might be involved in the modulation of PAI-1 expression in the hippocampus, but further investigation is required. Importantly, the increase in PAI-1 in CFSS rats was completely reversed by *C. butyricum* RH2 treatment but the decrease in tPA was not affected. As a result, *C. butyricum* RH2 exerted anxiolytic effect more likely by the inhibition of PAI-1 expression in the hippocampus after stress, at least in part. The up-regulation of PAI-1 has been implicated in the stress-induced behavioral disorders in animals and patients (Tang et al., 2015; Jiang et al., 2016; Han et al., 2019). Collectively, it was proposed that the CFSS-induced PAI-1 expression might be involved in CFSS-provoked mood abnormalities through inhibition of tPA cleavage activity, resulting in compromised maturation of BDNF and accumulated proBDNF. Additionally, PAI-1 may also cause depression by a mechanism independent of tPA and BDNF pathway but associated with impaired serotonin and dopamine metabolism (Party et al., 2019). Whether the disruption of serotonin and dopamine metabolism mediates the effects of PAI-1 in CFSS rats needs to be examined in more details in future studies. These findings suggest that targeting PAI-1 could be an innovative strategy for the development of new drugs to counter the effects of stress.

Gut dysbiosis has been implicated in modulating peripheral PAI-1 levels. The consumption of probiotic yogurt resulted in a significant reduction in the level of PAI 1 in patients with metabolic syndrome (Rezazadeh et al., 2019). A study by Gomez-Arango demonstrates a close association of the decrease of butyrate-producing bacteria and butyrate production in the gut microbiota with the increase in circulating PAI-1 levels in obese pregnant women with higher blood pressure (Gomez-Arango et al., 2016). However, few studies have been carried out to link the gut microbiota to PAI-1 in the brain. In this study, *C. butyricum* RH2 supplementation by gastric gavage reversed the increase of PAI-1 expression in the hippocampus of CFSS rats, providing the first evidence that brain PAI-1 expression can be modulated by gut microbiome. Further investigations are needed to determine the causality and to explore the potential mechanisms whereby *C. butyricum* RH2 may exert direct or indirect actions on PAI-1 expression in the hippocampus via butyrate or other metabolites.

In conclusion, a 2-week CFSS led to partial anxiety- or depression-like behaviors and also mild cognitive impairment. *C. butyricum* RH2 treatment reversed the CFSS-induced symptoms. This action may be mediated by changing BDNF and proBDNF imbalance and also be attributable to the inhibition of PAI-1 expression in the brain independent of BDNF and probDNF pathway.

### 4.1 Limitations

Effects of *C. butyricum* RH2 treatment on the CFSS-induced gut dysbiosis remain to be investigated. Furthermore, correlation of *C. butyricum* RH2-induced downregulation of PAI-1 in the hippocampus of CFSS rats with relevant behavior amelioration needs to be analyzed.

## Data Availability

The original contributions presented in the study are included in the article/Supplementary Material, further inquiries can be directed to the corresponding authors.
